# Behaviour change in perinatal care practices among rural women exposed to a women's group intervention in Nepal [ISRCTN31137309]

**DOI:** 10.1186/1471-2393-6-20

**Published:** 2006-06-15

**Authors:** Angie Wade, David Osrin, Bhim Prasad Shrestha, Aman Sen, Joanna Morrison, Kirti Man Tumbahangphe, Dharma S Manandhar, Anthony M de L Costello

**Affiliations:** 1 Centre for Paediatric Epidemiology and Biostatistics, Institute of Child Health, University College London, 30 Guilford Street London WC1N 1EH, UK; 2 International Perinatal Care Unit, Institute of Child Health, University College London, 30 Guilford Street London WC1N 1EH, UK; 3 Mother and Infant Research Activities (MIRA), GPO Box 921, Kathmandu, Nepal

## Abstract

**Background:**

A randomised controlled trial of participatory women's groups in rural Nepal previously showed reductions in maternal and newborn mortality. In addition to the outcome data we also collected previously unreported information from the subgroup of women who had been pregnant prior to study commencement and conceived during the trial period. To determine the mechanisms via which the intervention worked we here examine the changes in perinatal care of these women. In particular we use the information to study factors affecting positive behaviour change in pregnancy, childbirth and newborn care.

**Methods:**

Women's groups focusing on perinatal care were introduced into 12 of 24 study clusters (average cluster population 7000). A total of 5400 women of reproductive age enrolled in the trial had previously been pregnant and conceived during the trial period.

For each of four outcomes (attendance at antenatal care; use of a boiled blade to cut the cord; appropriate dressing of the cord; not discarding colostrum) each of these women was classified as BETTER, GOOD, BAD or WORSE to describe whether and how she changed her pre-trial practice. Multilevel multinomial models were used to identify women most responsive to intervention.

**Results:**

Among those not initially following good practice, women in intervention areas were significantly more likely to do so later for all four outcomes (OR 1.92 to 3.13). Within intervention clusters, women who attended groups were more likely to show a positive change than non-group members with regard to antenatal care utilisation and not discarding colostrum, but non-group members also benefited.

**Conclusion:**

Women's groups promoted significant behaviour change for perinatal care amongst women not previously following good practice. Positive changes attributable to intervention were not restricted to specific demographic subgroups.

## Background

Maternal and newborn mortality rates remain unacceptably high in the developing world. Most births and newborn deaths occur outside health facilities, so behaviour change in relation to home care practices and care-seeking behaviour is an essential component of any strategy to reduce deaths. We reported previously a cluster randomised controlled trial of the effects of participatory women's groups on neonatal outcomes in rural Nepal[1]. The trial intervention was a woman facilitator (who was not a trained health worker) within each area paid to instigate and guide women's groups focused on care in the perinatal period. The trial showed significant falls in neonatal (30%) and maternal mortality (78%), and appeared to be cost effective[2].

Married women of reproductive age (15–49 years) living in the study areas at the time of study inception were eligible. Before the trial started, each eligible woman was asked about her most recent pregnancy. If this resulted in a stillbirth, infant care practices were asked in respect of the most recent live birth. Information as to who was present at the birth and whether it took place in an institution was recorded. In particular it was ascertained whether there was a skilled attendant at the birth. The woman was asked whether she had attended antenatal care, which implement was used to cut the cord, what was applied to the cord after it was cut (the criterion for cleanliness was that either nothing or antiseptic was used) and whether or not she had discarded colostrum before starting to breastfeed, a practice distinct from discarding the foremilk at each feed. The evidence base for deciding which care practices are beneficial for good perinatal outcome is limited[3]. However, the practices recorded within the trial (antenatal care, skilled birth attendance, measures of cleanliness and good breastfeeding practice) have long been accepted as important[4-8].

After the baseline interview each woman became a member of the closed cohort who were randomised within village development committee areas (VDCs) and followed prospectively. In the original trial the efficacy of women's groups was measured for all women living within intervention areas (compared with control areas), even though many did not attend groups. The use of pre-trial pregnancy data allowed us to investigate the precise patterns of behaviour change within individual women and subgroups of women. Some women, in both arms of the trial, did not have the capacity for positive change attributable to intervention because they followed good practice in a pre-trial pregnancy. For women who did not follow good practice before the trial, our study gives us greater insight into factors affecting positive behaviour change, such as group membership, socioeconomic status, ethnicity and maternal age. The subset of women used for these analyses had by definition a pre-trial pregnancy and the results are not necessarily generalisable to women whose first pregnancy occurred in the trial.

## Methods

Details of the original trial are reported elsewhere[9,10]. Briefly, 24 cluster units comprising village development committee areas (VDCs) – existing geopolitical units of population about 7000 – were placed into 12 matched pairings of similar topography, ethnicity and population densities. One VDC area of each matched pair was randomly assigned to receive the intervention and the other formed a control. All eligible women were identified and details of pregnancies, births and deaths were recorded prospectively for 33 months.

The analysis includes women who had reported a previous pregnancy and who had a subsequent pregnancy during the surveillance period. Twin pregnancies were included only once in the dataset as the process outcomes under consideration mostly related to the delivery or woman at that time rather than the individual child. Repeated pregnancies were included in the analysis with the appropriate clustering to account for within-woman correlation of outcomes and responses.

### Statistical analysis

The practices undertaken in each trial pregnancy were compared with those practices a woman had reported in her pre-trial pregnancy. Each practice was classified for each pregnancy as:

1) BETTER – lack of good practice in the preceding pregnancy followed by good practice in the trial pregnancy.

2) GOOD – good practice in both preceding and trial pregnancies.

3) BAD – lack of good practice in both preceding and trial pregnancies.

4) WORSE – good practice in the preceding pregnancy but not in the trial pregnancy.

We fitted multilevel multinomial models, taking into account the pairing of VDC area clusters, the clustering of women within VDC areas and households, and the correspondence between repeat prospective pregnancies in the same woman, to the 4-category outcomes. Multinomial models were preferred to logistic regressions of the trial practices corrected for pre-trial behaviour since they distinguished between changes from bad to good or from good to bad practice.

Multinomial models are extensions of logistic models. Associations between the outcomes and various features of the women are quantified and presented as coefficients for the ratios falling into the BETTER category relative to the other categories. This representation of the model results was chosen as being the easiest to interpret clinically. For all 3 ratios thus obtained, larger values were associated with more favourable outcome. All coefficients are presented with 95% confidence intervals adjusted for the clustered nature of the data. For each feature, separate coefficients are given to quantify the ratios:

1) BETTER relative to GOOD – quantifies the extent to which women following good practice in the trial pregnancy were doing so as a result of positive change (as opposed to continuing the good practices they had adopted pre-trial).

2) BETTER relative to BAD – quantifies the extent to which women who were following bad practice in the pre-trial pregnancy improved their practice within the trial. This coefficient is of particular interest as it describes the extent to which opportunities for positive change were taken.

3) BETTER relative to WORSE – quantifies the extent to which those women who changed practice made a positive, as opposed to negative, change.

A series of multilevel multinomial models were fitted to each of the process variables. Firstly, models were used to quantify differences in patterns of change between control and intervention clusters and the additional effect of attending a women's group for women within intervention clusters.

Secondly, a series of models were fitted to investigate whether the effect of intervention varied between women of differing ages, literacy levels and education, or between those living within households of differing ethnicity, assets or food sufficiency. For each of these demographic variables, a model which incorporated the demographic variable, a variable representing intervention status, and a term for the interaction between these two variables was used. The intervention and demographic variables were independently significantly associated with outcomes in all models. The coefficients for the fitted interaction terms showed which groups of women were most likely to respond to intervention and these are presented. Coefficients greater than 1 indicate that the women in the intervention clusters within that demographic subgroup had a more favourable distribution compared to the baseline category which was over and above any increase in favourable practices that could be attributed to intervention across all subgroups. Coefficients less than 1 are associated with a less favourable response for that demographic subgroup of women in the intervention compared with control clusters.

#### Ethics and consent

The study was registered as an International Standard Randomised Controlled Trial, number ISRCTN31137309. It was approved by the Nepal Health Research Council and the ethical committee of the Institute of Child Health and Great Ormond Street Hospital for Children, and was conducted in collaboration with His Majesty's Government Ministry of Health, Nepal. The aims and design of the trial were discussed at both national and local meetings, after which consent to cluster involvement was given by chairpersons of VDC areas and the Makwanpur district development committee. Women who chose to participate in the study gave oral consent, were free to decline to be interviewed at any time, and the information they provided remained confidential.

## Results

Of the women for whom information regarding a previous pregnancy had been recorded, 4929 had one further pregnancy during the trial surveillance period, 228 had 2 pregnancies and 5 had 3 pregnancies. Hence, there were a total of 5400 within-trial pregnancies from women with retrospectively recorded information.

Most women delivered at home (93%), without a trained attendant (92%) or any government health personnel present (90%), in either the preceding or study pregnancy. The sentinel care practices of antenatal care uptake, use of a clean blade to cut the umbilical cord, appropriate dressing of the cord and feeding of colostrum to the baby were more variably followed. Table 1 shows the demographic breakdown of the women and the extent to which good practice was being followed prior to commencement of the study.

**Table 1:** Practices in pre-trial pregnancies according to demographic variables

	Number following good practice pre-trial (% of total)			
	Antenatal care attendance	Boiling the blade	Appropriate dressing of cord	Not discarding colostrum
	n = 5373 (%)	n = 5216 (%)	N = 5216 (%)	n = 5120 (%)

**Household**				
Ethnicity:				
Tamang	757 (21.2)	648 (18.6)	2654 (76.3)	1543 (45.3)
Brahmin-Chhetri	551 (67.0)	512 (65.0)	601 (76.3)	535 (68.8)
Magar	103 (40.9)	83 (33.5)	187 (75.4)	151 (61.9)
Other	212 (29.2)	258 (36.8)	526 (74.9)	407 (59.0)

No assets listed	651 (22.1)	634 (22.2)	2175 (76.0)	1333 (47.5)
Clock, radio, iron, bicycle	569 (31.9)	517 (29.8)	1305 (75.2)	896 (52.6)
More costly appliances	403 (62.5)	350 (56.6)	488 (79.0)	407 (66.8)

**Mother**				
Illiterate	704 (18.6)	713 (19.3)	2816 (76.3)	1628 (45.0)
Reads with difficulty	320 (45.3)	285 (41.5)	522 (76.1)	418 (62.1)
Reads with ease	599 (68.5)	503 (60.0)	630 (75.2)	590 (71.3)

No formal education	949 (21.8)	933 (22.1)	3219 (76.2)	1959 (47.3)
Primary schooling only	395 (57.2)	325 (48.7)	503 (75.3)	424 (64.4)
Secondary or higher	279 (82.8)	243 (75.7)	246 (76.6)	253 (79.3)

TOTAL	1623 (30.2)	1501 (28.8)	3968 (76.1)	2636 (51.5)

	Median (Interquartile range) for those following good (G) and bad (B) practice retrospectively:			

**Household**				
Number of months with	G: 10 (7,12)	G: 12 (7,12)	G: 10 (7, 12)	G: 10 (7, 12)
sufficient food	B: 9 (7,12)	B: 9 (7,12)	B: 9 (7, 12)	B: 10 (7, 12)

**Mother**				
Age (per additional year)	G: 22.2 (19.9, 26.2)	G: 22.7 (20.1, 27.0)	G: 24.6 (20.9, 29.8)	G: 24.0 (20.8, 28.8)
	B: 25.8 (21.6, 31.2)	B: 25.3 (21.3, 30.9)	B: 24.4 (20.9, 29.9)	B: 25.2 (21.1, 30.9)

Note: Numbers are less than 5400 for each outcome since some women did not have pregnancies that progressed to the stage for that outcome to be appropriate. For example, 280 of the eligible pregnancies did not result in a live birth of a surviving mother and hence the discarding of colostrum was only appropriate as an outcome for 5120 women.

Approximately three quarters of the women were appropriately dressing the cord initially and this proportion was fairly constant across demographic subgroups. Attendance at antenatal care, boiling of the blade and not discarding colostrum were all more prevalent amongst the more highly educated and literate women from wealthier households.

### The effect of being in an intervention VDC

Table 2 shows the percentages of pregnancy pairings falling into each of the 4 categories (BETTER, GOOD, BAD, WORSE) for the 4 outcomes for women in intervention and control arms of the trial.

**Table 2:** Behaviour change over time between pre-trial and trial pregnancies for four perinatal care practices.

		Antenatal care attendance	Boiling the blade	Appropriate dressing of cord	Not discarding colostrum
	Intervention	n = 2535	n = 2454	n = 2454	n = 2409
	Control	n = 2838	n = 2762	n = 2762	n = 2711
% BETTER	Intervention	19.1	21.8	17.9	29.7
	Control	16.6	12.1	16.3	23.2

% GOOD	Intervention	36.0	32.5	63.2	41.7
	Control	12.5	12.8	56.7	34.7

% BAD	Intervention	36.8	39.2	4.2	17.5
	Control	65.6	68.1	9.3	26.5

% WORSE	Intervention	8.2	6.4	14.7	11.2
	Control	5.2	7.0	17.8	15.5

TOTAL (%)	Intervention	100	100	100	100
	Control	100	100	100	100

Intervention/control comparisons : Odds ratios (95% confidence interval)					

%BETTER/%BAD ratio *		2.04 (1.82, 2.27)	3.13 (2.78, 3.45)	2.44 (1.92, 3.13)	1.92 (1.69, 2.22)
%BETTER/%WORSE ratio*		0.73 (0.59, 0.91)	1.96 (1.59, 2.44)	1.33 (1.15, 1.54)	1.79 (1.52, 2.08)
%BETTER/%GOOD ratio*		0.40 (0.35, 0.46)	0.71 (0.61, 0.81)	0.99 (0.88, 1.10)	1.06 (0.95, 1.19)

*Results from multilevel multinomial models. The estimates and intervals are adjusted to take account of the correlations between pregnancies within the same women, women from the same household, households from the same VDC and VDCs within the same matched pair. All odds ratios are significantly different to 1. Coefficients greater than 1 indicate that the women in the intervention clusters had a more favourable distribution, those less than 1 are associated with a less favourable response for the women in the intervention compared with control clusters.

The percentage of women who were following good practice during their trial pregnancies can be obtained by adding together the percentages falling into the BETTER and GOOD categories. For each of the 4 outcomes a greater percentage of the women in the intervention clusters followed good practice during the trial.

Combining the BETTER and BAD categories gives the percentage of women who were following bad practice pre-trial (and hence had the capacity to change for the better). For all outcomes apart from the discarding of colostrum, control clusters had more women with that capacity than intervention clusters. The percentages of women who recalled discarding colostrum in their preceding births were approximately equal between control and intervention clusters. The percentages lying within the BAD category represent missed opportunities for positive change and there were consistently fewer women within intervention clusters falling into this category for each of the 4 outcomes.

Women who changed their practice between preceding and study pregnancies fell into the BETTER and WORSE categories. The percentage of women in the intervention clusters falling into the BETTER category was greater than the percentage in the WORSE category, showing that women were more likely to make a positive, as opposed to detrimental, change for all outcomes. Women in the control clusters were more likely to stop, as opposed to start, appropriate dressing of the cord, but otherwise their changes were similarly more likely to be in a positive direction.

The differences between women in the intervention and control VDCs are further quantified by the fitting of multinomial models to the 4 outcomes with intervention status as a predictor. The coefficients and confidence intervals are given for the BETTER/BAD and BETTER/WORSE ratios. These are all significantly different to 1. For all four practices women who were initially following bad practice were significantly more likely to change to good practice if they lived in an intervention VDC (BETTER/BAD ratios). For example, women who did not attend antenatal care in preceding pregnancies were more than twice as likely to do so during the study period if they lived in an intervention area (odds ratio 2.04 95% ci (1.82, 2.27 times)). Of the women who changed practice these changes were significantly more likely to be in a positive direction for all outcomes except antenatal care attendance (BETTER/WORSE ratio).

Women attending antenatal care and/or using a boiled blade to cut the cord in pregnancies falling within the study period were significantly less likely to be doing so as a result of a positive change in practice if they lived in an intervention VDC (BETTER/GOOD ratios). These results are not unexpected given the larger percentages of women within the intervention VDCs following good practice for these outcomes pre-trial.

### The independent effect of attending a women's group

About one in twelve married women of reproductive age, and about one third of newly pregnant women in intervention clusters attended the women's groups. There were few differences between the percentages of women who did and did not attend women's groups falling into each of the 4 categories.

The effect of attending a group over and above the improvements attributable to living within an intervention area was greatest for antenatal care attendance. The percentages of women who attended the groups falling into the BETTER, GOOD, BAD and WORSE categories were 22.3, 37.3, 33.7 and 6.7 respectively, compared to 17.8, 34.9, 38.9 and 9.1 of those within intervention VDCs who did not attend groups. Hence, a larger percentage of those attending the women's groups improved their practice (22.3 vs 17.8%) or maintained previous good practice (37.3 vs 34.9%). The significantly lower odds of making a positive as opposed to negative change (BETTER/WORSE ratio) in the intervention VDCs were counter-acted in the subgroup who attended the women's groups. The women who attended the groups were significantly more likely to make positive changes than non-attending women within intervention VDCs (BETTER/WORSE ratio 1.77 (1.30, 2.40)). Similarly, the women within intervention VDCs who attended the groups but did not attend antenatal care in their previous pregnancies were significantly more likely to start doing so than the women within those same VDCs who did not attend (BETTER/BAD ratio 1.51 (1.28, 1.79)). This difference was additional to the 2.04 fold increase seen in the intervention VDCs overall. The BETTER/GOOD ratio for attenders vs non-attenders was also significant (1.22 (1.04, 1.45)). Women attending the groups were significantly more likely to make positive changes compared to non-attending women in the same VDCs with respect to discarding colostrum (BETTER/WORSE ratio 1.03 (1.01, 1.06)) and there was some evidence that if they were discarding colostrum previously they were more likely to stop doing so (BETTER/BAD ratio 1.02 (1.00, 1.04)). There were no other significant differences.

### Were specific subgroups of women with the capacity for positive change more likely to respond to intervention?

Table 3 shows the increase in the BETTER/BAD ratios for the intervention group compared to the women in control areas. Values greater than 1 indicate that the intervention was more successful in those subgroups of women relative to the baseline demographic category. Significant differences in the effects of intervention on the four process outcomes were not consistent across demographic subgroups.

**Table 3:** Coefficients and 95% confidence intervals for the extent to which women in the intervention VDCs, relative to women in the control VDCs, within different demographic subgroups were more (or less) likely to make a positive change, relative to those in the baseline subgroup, if they were not initially following good practice (%BETTER/%BAD ratio)

	Antenatal care attendance N = 5373	Boiling the blade prior to cord cutting n = 5216	Appropriate dressing of the cord n = 5216	Not discarding colostrum n = 5120
**Household:**				
Ethnicity:				
Tamang	1	1	1	1
...Brahmin-Chhetri	1.41 (0.98, 2.04)	**0.43 (0.28, 0.68)**	1.11 (0.55, 2.22)	1.16 (0.70, 1.89)
Magar	**4.17 (2.27, 7.69)**	0.95 (0.54, 1.67)	0.38 (0.12, 1.27)	**2.50 (1.01, 6.25)**
Other	**1.85 (1.23, 2.86)**	0.74 (0.52, 1.06)	**8.33 (1.92, 33.33)**	**1.61 (1.04, 2.50)**

No assets listed	1	1	1	1
Clock, radio, iron, bicycle	0.84 (0.65, 1.08)	0.96 (0.75, 1.23)	1.10 (0.65, 1.82)	1.02 (0.76, 1.37)
More costly appliances	0.73 (0.48, 1.11)	1.06 (0.70, 1.59)	1.92 (0.85, 4.35)	0.77 (0.44, 1.33)

Number of months with sufficient food	1.01 (0.97, 1.05)	0.97 (0.93, 1.01)	**1.10 (1.01, 1.20)**	**1.05 (1.00, 1.10)**

**Mother:**				
Age (per additional year)	0.99 (0.97, 1.01)	1.00 (0.98, 1.02)	**0.93 (0.91, 0.97)**	1.00 (0.99, 1.02)

Illiterate	1	1	1	1
Reads with difficulty	**0.59 (0.41, 0.83)**	1.08 (0.76, 1.54)	1.09 (0.54, 2.17)	0.67 (0.42, 1.04)
Reads with ease	0.73 (0.50, 1.08)	**0.68 (0.46, 1.00)**	0.89 (0.44, 1.79)	1.04 (0.64, 1.69)

No formal education	**1**	1	1	1
Primary schooling only	**0.57 (0.40, 0.83)**	**0.42 (0.29, 0.61)**	**1.47 (1.08, 2.04)**	1.28 (0.81, 2.04)
Secondary or higher	**0.25 (0.11, 0.58)**	0.53 (0.21, 1.35)	0.92 (0.57, 1.47)	0.50 (0.13, 1.92)

(Results from multilevel multinomial models. The estimates and intervals are adjusted to take account of the correlations between pregnancies within the same women, women from the same household, households from the same VDC and VDCs within the same matched pair. Significant differences are shown in **bold**.)

### Were women who changed practice more likely to do so positively if they were from specific subgroups?

The extent to which women made positive, as opposed to negative, changes in practice is quantified by the BETTER/WORSE model coefficients. Patterns were not consistent (Table 4) but they were based on the smallest groups (Table 2). Women from households with more assets within intervention VDCs were significantly more likely to make a positive change to dressing the cord but a negative change with respect to the treatment of colostrum. It was the older women, those who were less literate and the less well educated who were significantly more likely to have stopped, as opposed to started, discarding colostrum if they lived in intervention, as opposed to control, VDCs.

**Table 4:** Coefficients and 95% confidence intervals for the extent to which women in the intervention VDCs, relative to women in the control VDCs, within different demographic subgroups were more (or less) likely to make positive as opposed to negative changes, relative to those in the baseline subgroup, (%BETTER/%WORSE ratio)

	Ante-natal care attendance n = 5373	Boiling the blade prior to cord cutting n = 5216	Appropriate dressing of the cord n = 5216	Not discarding colostrum n = 5120
**Household:**				
Ethnicity:				
Tamang	1	1	1	1
...Brahmin-Chhetri	**0.58 (0.39, 0.88)**	0.76 (0.45, 1.28)	**1.85 (1.14, 2.94)**	0.79 (0.47, 1.35)
Magar	0.67 (0.39, 1.15)	1.30 (0.37, 4.55)	0.65 (0.32, 1.30)	**3.57 (1.32, 10.00)**
Other	0.83 (0.59, 1.16)	0.59 (0.29, 1.19)	**2.38 (1.52, 3.85)**	**2.27 (1.33, 3.85)**

No assets listed	1	1	1	1
Clock, radio, iron, bicycle	0.92 (0.57, 1.47)	1.15 (0.71, 1.85)	1.37 (0.98, 1.89)	0.99 (0.68, 1.43)
More costly appliances	1.92 (0.97, 3.85)	0.89 (0.44, 1.82)	**2.33 (1.45, 3.85)**	**0.36 (0.18, 0.70)**

Number of months with sufficient food	0.97 (0.89, 1.05)	0.98 (0.89, 1.06)	1.02 (0.96, 1.08)	0.99 (0.93, 1.05)

**Mother:**				
Age (per additional year)	**0.96 (0.93, 1.00)**	1.01 (0.97, 1.04)	0.98 (0.96, 1.01)	**1.04 (1.01, 1.06)**

Illiterate	1	1	1	1
Reads with difficulty	0.91 (0.51, 1.64)	1.20 (0.66, 2.22)	1.03 (0.62, 1.72)	**0.31 (0.18, 0.52)**
Reads with ease	1.54 (0.89, 2.63)	1.19 (0.69, 2.04)	1.47 (0.97, 2.22)	0.61 (0.36, 1.05)

No formal education	1	1	1	
Primary schooling only	1.08 (0.60, 1.96)	1.03 (0.56, 1.92)	1.11 (0.84, 1.47)	**0.49 (0.27, 0.87)**
Secondary or higher	2.04 (0.93, 4.55)	0.99 (0.46, 2.17)	0.97 (0.95, 1.00)	0.79 (0.34, 1.85)

(Results from multilevel multinomial models. The estimates and intervals are adjusted to take account of the correlations between pregnancies within the same women, women from the same household, households from the same VDC and VDCs within the same matched pair. Significant differences are shown in **bold**.)

### Were women from specific subgroups who followed good practice during the trial more likely to be doing so as a result of a positive change?

These differences are quantified in Table 5 (BETTER/GOOD ratios). This table is presented for completeness. However, it is of the least clinical interest due to the dependence on the variability between groups of the percentages who show no changes but continue good practice throughout.

**Table 5:** Coefficients and 95% confidence intervals for the extent to which women in the intervention VDCs, relative to women in the control VDCs, within different demographic subgroups who were following good practice during the study period were more (or less) likely to be doing so as a result of a positive change in practice (%BETTER/%GOOD ratio)

	Ante-natal care attendance n = 5373	Boiling the blade prior to cord cutting n = 5216	Appropriate dressing of the cord n = 5216	Not discarding colostrums n = 5120
**Household:**				
Ethnicity:				
Tamang	1	1	1	1
...Brahmin-Chhetri	0.79 (0.44, 1.41)	**4.35 (2.94, 6.67)**	**1.52 (1.09, 2.08)**	**1.72 (1.25, 2.38)**
Magar	0.71 (0.22, 2.22)	**5.26 (2.70, 10.0)**	**2.44 (1.37, 4.17)**	**3.33 (1.92, 5.88)**
Other	1.49 (0.68, 3.33)	**2.63 (1.69, 4.00)**	0.91 (0.65, 1.28)	**1.45 (1.02, 2.04)**

No assets listed	1	1	1	1
Clock, radio, iron, bicycle	1.28 (0.93, 1.75)	**1.61 (1.16, 2.22)**	**1.28 (1.01, 1.64)**	1.12 (0.88, 1.45)
More costly appliances	1.33 (0.89, 2.00)	**2.13 (1.43, 3.23)**	**2.94 (2.04, 4.35)**	1.19 (0.82, 1.72)

Number of months with sufficient food	**1.05 (1.00, 1.11)**	**1.11 (1.05, 1.19)**	**1.06 (1.02, 1.11)**	0.95 (0.91, 1.00)

**Mother:**				
Age (per additional year)	1.01 (0.99, 1.04)	1.00 (0.98, 1.03)	0.99 (0.98, 1.01)	1.01 (0.99, 1.03)

Illiterate	1	1	1	1
Reads with difficulty	0.94 (0.63, 1.41)	1.41 (0.94, 2.13)	**1.67 (1.18, 2.33)**	0.99 (0.71, 1.39)
Reads with ease	**1.56 (1.10, 2.22)**	**1.59 (1.12, 2.22)**	**1.56 (1.14, 2.13)**	**1.47 (1.06, 2.04)**

No formal education	1	1	1	1
Primary schooling only	1.35 (0.94, 1.96)	1.06 (0.74, 1.54)	1.01 (0.87, 1.18)	1.35 (0.96, 1.92)
Secondary or higher	1.54 (0.91, 2.56)	**1.89 (1.14, 3.13)**	1.15 (0.93, 1.41)	1.59 (0.93, 2.70)

(Results from multilevel multinomial models. The estimates and intervals are adjusted to take account of the correlations between pregnancies within the same women, women from the same household, households from the same VDC and VDCs within the same matched pair. Significant differences are shown in **bold**.)

## Discussion

Within a large scale trial of a community group intervention, women were followed prospectively to document patterns of behaviour change for perinatal care. This helps to understand how primary trial outcomes may be explained by changes in the practices of individuals within the communities. Of the 6380 women who became pregnant and were included in the main trial analyses, a subset of 5162 (77.3%) had a pregnancy pre-trial with which to compare their trial pregnancy behaviour. Within this subgroup we have investigated the changes for women undergoing their second or subsequent pregnancies. The findings cannot be extrapolated to women in their first pregnancies.

As expected, there were strong relationships between past and present behaviour. Those who followed good practice in previous pregnancies were likely to do so again, regardless of whether they were allocated to the intervention or control arm of the trial. Having a skilled birth attendant is known to be an important indicator of outcome. Less than 1 in 12 of the women had such a person present at either their pre-trial or any trial pregnancy. The numbers therefore were too low to investigate any impact the intervention may have had on improving skilled birth attendance. However, it was possible to investigate changes in other factors known to be important: antenatal care, cleanliness of blade and cord, and discarding of colostrum. The intervention effectively promoted significant change in all four care behaviours amongst the group of women not previously following good practice (Table 2). Positive changes in antenatal care attendance and the discarding of colostrum were more likely to be made by women who attended the groups, but behaviour change in hygienic cutting and dressing is observed generally in the intervention areas. The lack of uniform relationship between group attendance and outcome was expected. The presence of groups in an area has a wider impact than merely on the women who attend. In our study only 8% of married women of reproductive age joined our groups, but 37% of newly pregnant women attended at least once. Whilst group members showed a greater tendency to positive behaviour change than non-group members, this effect is unlikely to explain the overall improved behaviour change in intervention versus control clusters. Our data provides evidence that the activities and existence of the group stimulate wider behaviour change in their communities. The group intervention is a dynamic process that is uniform only in its participatory method, thus further study is necessary to explore these processes of behaviour change. We hoped to bring about behaviour change by giving women and grandmothers the knowledge they need to make informed choices, and by creating favourable social conditions, and an enabling environment in which they could take these decisions[11-13]. Preliminary analysis of qualitative data, and the data presented here suggest that this has been the case in the intervention areas.

Most of the responses to intervention were positive. Significantly greater percentages of women in intervention VDCs who were following bad practice pre-trial stopped doing so after the commencement of the women's groups. It was surprising that significantly more of the intervention area women stopped as opposed to started attending antenatal care compared to the women within control VDCs. This difference was mostly attributable to the greater proportion of women in intervention VDCs who stopped attending. For the other 3 practices a greater percentage of control women stopped previous good practice and for all 4 practices there was a lower percentage who started. It is possible that the women in the intervention VDCs saw women's groups as a replacement for antenatal care. This potentially detrimental effect of the intervention requires further investigation, perhaps via the use of focus groups in similar future initiatives.

Since allocation was random, we would not expect baseline differences between women in control and intervention VDCs. Despite similar mortality rates at baseline[1], some differences in practices were found in the subgroup with a previous pregnancy reported here. In particular, women within intervention VDCs were more likely to have attended antenatal care (44.2% intervention, 17.7% control) and to have boiled the blade (38.9% and 19.8% respectively) in their pre-trial pregnancies. The analyses presented in this paper show that multigravid women in intervention VDCs were significantly more likely to continue or begin good practices after accounting for baseline differences. These significant differences in behaviour within this subgroup of just over three-quarters of the women who fell pregnant within the trial period are compatible with the reductions in major outcomes found within the trial.

We have presented secondary analyses of the dataset. Many comparisons are presented and these are meant to be interpreted in unison and with the main outcome analyses. The study was not originally designed to detect subgroup differences and the results should not be interpreted as though they were primary objectives. What we have aimed to do is to identify patterns that might be clinically relevant and informative to future studies. We have not identified any major consistent patterns, a finding which is itself of interest. Having identified significant differences which could be attributed to the intervention[1], this analysis investigates the modes via which those differences may have been achieved. We would expect to observe differences in process outcomes since these are known to be related to mortality outcomes. The finding that the intervention was associated with increased uptake of good practices in those previously not following them is both important and as expected: in this paper we attempt to quantify the degree of difference. It is also important to note that no tendency for the intervention to target only subgroups of privileged or non-privileged women was found. Prior to performing these analyses we had no notion of the direction that any intervention bias might fall.

If women were already following good practice, the capacity for the women's groups to effect positive change was limited. It was important that those following good practices continued to do so. Therefore, we have considered all patterns of change and how they related to features of the mother, the household in which she lived and whether or not she resided in an intervention area. Four pre-trial practices were found to have a large capacity for positive change and for there to have been significant alterations during the study period. The women's groups discussed the prevention of neonatal deaths, home care practices that might help, and the use of health services for either routine or emergency care. The issues of antenatal care, the use of clean cord-cutting implements, avoidance of unhygienic dressings and the benefits of colostrum feeding arose as subjects of discussion on many occasions. These issues could, and were, easily translated into specific actions.

It was clear that the less educated and illiterate women were less likely to be following good practice initially. Although these women were significantly targeted by the intervention for some outcomes, the differences were not uniform. There were benefits across all of the demographic subgroups of women.

## Conclusion

In conclusion, peer-education and empowerment of women through women's groups has positive effects on perinatal care practices for women in their second or subsequent pregnancies. Both group members and non-group members in the locality benefit from this intervention.

## Competing interests

The author(s) declare that they have no competing interests.

## Authors' contributions

AW drafted the initial paper, helped design the original study, devised the analysis plan, conducted the analyses and provided an initial interpretation. DO, BPS, AS, JM, KMT, DSM and AMC devised and designed the study, assisted in the interpretation of the data and commented on multiple drafts of the paper. All authors read and approved the final submission.

## Funding detail

The study was funded by the Department for International Development of the United Kingdom, with important support from the Division of Child and Adolescent Health, World Health Organization, Geneva, the United Nations Children's Fund (UNICEF), Nepal, and the United Nations Fund for Population Activities (UNFPA), Nepal. The Department for International Development can accept no responsibility for any information provided or views expressed.

## Pre-publication history

The pre-publication history for this paper can be accessed here:


